# 宏基因组学测序技术诊断单倍体造血干细胞移植后腺病毒感染6例临床分析

**DOI:** 10.3760/cma.j.issn.0253-2727.2022.10.013

**Published:** 2022-10

**Authors:** 艳珺 吴, 峰 陈, 晔 赵, 彦明 张, 俊杰 曹, 国强 林, 婷敬 王, 晶 夏, 晓文 唐, 胜利 薛, 正明 金, 德沛 吴

**Affiliations:** 1 苏州大学附属第一医院血液科，江苏省血液研究所，苏州大学造血干细胞移植研究所，苏州 215006 First Affiliated Hospital of Soochow University, Jiangsu Institute of Hematology, Institute of Blood and Marrow Transplantation, Soochow University, Suzhou 215006, China; 2 江苏省淮安市第二人民医院血液科，淮安 223002 Department of Hematology, Huai'an Second People's Hospital, Huai'an 223002, China; 3 宁波大学附属人民医院血液科，宁波 315040 Department of Hematology, Yinzhou People's Hospital, Ningbo 315040, China

单倍体造血干细胞移植（Haplo-HSCT）近年在国内异基因移植（allo-HSCT）中已占比60％，成为最主要的移植模式[1]。基于“北京方案”的Haplo-HSCT疗效确切，但免疫重建延迟也相应增加了机会性感染风险。近期国内外陆续有allo-HSCT后发生腺病毒（ADV）感染的病例报道，多数缺乏早期诊断，且预后不良[2]–[4]。新兴的宏基因组学测序技术（mNGS）为免疫缺陷患者的少见、罕见病原学感染事件提供了新的诊断线索和检验效能的补充。我们对国内3个移植中心Haplo-HSCT后通过mNGS诊断的6例ADV感染患者进行了回顾性观察分析，现报道如下。

## 病例与方法

1. 病例：2019年1月至2021年8月，在苏州大学附属第一医院（3例）、江苏省淮安市第二人民医院（2例）、宁波大学附属人民医院（1例）接受Haplo-HSCT并通过mNGS确诊ADV感染的6例患者。急性白血病5例，再生障碍性贫血1例。白血病诊断符合WHO 2016造血和淋巴组织肿瘤分类标准，再生障碍性贫血诊断符合《再生障碍性贫血诊断与治疗中国专家共识（2017年版）》[5]。

2. Haplo-HSCT：预处理方案：5例采用改良白消安（Bu）-环磷酰胺（Cy）方案，1例为全身照射（TBI）-Cy方案，均联合抗胸腺细胞球蛋白（ATG）。移植物来源：6例均为G-CSF动员后的供者骨髓+外周血造血干细胞，其中2例联合第三方脐血干细胞。移植物抗宿主病（GVHD）预防：钙调磷酸酶抑制剂+短程甲氨蝶呤+吗替麦考酚酯。其他相关并发症的预防均参考文献[6]方案进行。

3. mNGS检测方法：无菌留取外周血、脑脊液、尿液、肺泡灌洗液样本（至少3 ml）进行微生物mNGS检测，通过核酸提取、文库构建（末端修复、接头连接、PCR扩增、文库质量评估及文库测序池）、测序及数据的对比（NCBI微生物基因组数据库，fttp://ftp.ncbi.nlm.nih.gov/genomes/）和判读，确定检出的ADV序列数（Reads）。

4. ADV感染的判定：通过mNGS检测出的ADV序列数在3/100万及以上为可信[7]，同时患者同种样本的ADV-PCR复检结果为阳性[8]。

5. 随访：观察起点：患者首次诊断ADV感染。观察终点：患者ADV感染期间死亡或者连续两次检测结果提示ADV DNA转阴。患者临床资料均来自住院记录。

## 结果

6例Haplo-HSCT后ADV感染患者的主要临床资料见表1。男4例，女2例。ADV感染的危险因素包括6例患者均为Haplo-HSCT且预处理方案中包含ATG；3例患者有前驱激素耐药（SR）GVHD史，并接受CD25单抗、芦可替尼等二线治疗；2例患者外周血CD3^+^ T细胞计数<300个/µl；5例患者合并其他病原体感染，包括巨细胞病毒（CMV）、EB病毒（EBV）、尿多瘤病毒（BKV）等。4例患者诊断ADV血症前持续发热，影像学、常规检验无明显异常，经验性抗感染治疗无效。3例ADV脑炎患者确诊前均有突发的中枢神经系统表现，脑脊液常规检查均无明显异常。2例ADV肝炎患者，表现为肝功能急剧恶化，鉴别诊断困难，常规治疗无效。5例患者接受西多福韦（CDV）抗病毒治疗，4例接受静脉丙种免疫球蛋白（IVIg）治疗，1例接受CDV、IVIg、胸腺肽、低剂量的供者淋巴细胞输注（DLI）等治疗。截至随访终点，1例患者ADV DNA转为阴性，感染表现好转，但死于原发病复发；余5例均在ADV感染期间死亡。

**表1:** 6例宏基因组学测序技术诊断单倍体造血干细胞移植后腺病毒（ADV）感染患者的临床特征、ADV感染情况与转归

例号	性别	年龄（岁）	基础疾病	干细胞来源	危险因素				合并感染
					Ⅲ~Ⅳ度GVHD	应用CD25单抗/ATG	是否去T细胞	CD3^+^细胞<300个/µl	
1	男	23	AML伴髓外浸润	骨髓+外周血	无	ATG	否	否	BKV、EBV（治疗后均转阴）
2	男	31	ETP-ALL	骨髓+外周血	无	ATG	否	是	结核分枝杆菌、EBV、CMV（治疗后均转阴）
3	女	17	AML	骨髓+外周血+第三方脐血	无	ATG	否	是	CMV（治疗后转阴）
4	男	49	Ph^+^ ALL	骨髓+外周血	皮肤、肠道	ATG+CD25单抗	否	否	无
5	男	27	AML	骨髓+外周血	皮肤、肠道	ATG+CD25单抗	否	是	CMV、肺炎克雷伯杆菌（治疗后均转阴）
6	女	13	SAA	骨髓+外周血+第三方脐血	皮肤、肠道	ATG+CD25单抗	否	是	CMV（反复感染）、BKV（治疗后转阴）

例号	前驱表现	经验性治疗	ADV样本	影像学	诊断	诊断距移植时间	治疗	转归	确诊ADV感染至死亡时间
1	持续发热、血尿	经验性抗感染无效	外周血、尿ADV-B	CT：肾实质密度略高，伴左肾周渗出性改变	ADV尿道炎、ADV血症	99 d	CDV	ADV转阴，死于肿瘤复发	346 d
2	肝功能急剧恶化，凝血功能异常	保肝治疗无效	外周血ADV-C	腹部CT未见明显异常	ADV血症、ADV肝炎不除外	6个月	IVIg	ADV感染期间死亡	确诊当天死亡
3	癫痫、肝损伤、血便、发热	经验性抗感染及保肝治疗无效	外周血、脑脊液ADV-C	MRI：大脑及小脑多发异常信号	ADV血症、ADV脑炎、ADV肝炎不除外	108 d	CDV、IVIg、胸腺肽、DLI	ADV感染期间死亡	69 d
4	突发意识障碍	停CNI、血浆置换	脑脊液ADV-D	头颅MRI未见明显异常	ADV脑炎	78 d	CDV	ADV感染期间死亡	4 d
5	反复肺部感染，突发意识障碍	经验性抗感染无效	肺泡灌洗液ADV-C、脑脊液ADV-D	MRI：双侧海马及颞叶异常信号	ADV肺炎、ADV脑炎	25个月	CDV、IVIg	ADV感染期间死亡	21 d
6	反复发热	经验性抗感染无效	外周血ADV-C	MRI：颅内多发低密度灶；CT：双肺感染	ADV血症	80 d	CDV、IVIg	ADV感染期间死亡	153 d

注：AML：急性髓系白血病；ETP-ALL：早期前体急性T淋巴细胞白血病；SAA：重型再生障碍性贫血；GVHD：移植物抗宿主病；ATG：抗胸腺细胞球蛋白；BKV：尿多瘤病毒；EBV：EB病毒；CMV：巨细胞病毒；CNI：钙调磷酸酶抑制剂；CDV：西多福韦；IVIg：静脉注射免疫球蛋白；DLI：供者淋巴细胞输注

## 讨论

allo-HSCT患者发生病毒感染（活化或疾病），不限于最常见的CMV、EBV等人类疱疹病毒属（HHV）病毒，已报道可感染超过20余种病毒（包括RNA病毒）[9]。ATG、抗B细胞/T细胞单抗、CD3-CD19双特异性抗体、嵌合抗原受体T细胞（CAR-T细胞）及治疗中某些高效免疫抑制剂（如芦可替尼等）的应用，一定程度上会增加allo-HSCT患者病毒感染风险。除CMV、EBV外，目前国内多数移植中心尚未开展其他病毒的常规监测。近年我中心通过mNGS技术，结合其他检验及临床特征，确诊数例Haplo-HSCT后ADV感染病例，汇同另两家中心病例，进行多中心、回顾性观察分析。这也是迄今国内HSCT后ADV感染最大病例数的临床报道。

mNGS是一种基于二代高通量测序平台，快速获取检测样本中的核酸序列（DNA及RNA），经与数据库比对，从而获取样本中微生物种属水平的种类和比例的检测技术。自2014年Wilson等[10]首次报道了通过mNGS确诊钩端螺旋体脑炎1例，mNGS逐渐广泛应用于临床。近期我中心对149例allo-HSCT后疑诊感染者，对比分析mNGS与传统微生物学检验，显示前者可显著提高allo-HSCT后疑难感染的检验效能[11]。本研究中6例ADV确诊病例，前驱表现为不明原因的持续发热、急性肝损伤、肺炎、中枢神经系统异常等，常规检验无法明确病因，经验性治疗无效，经mNGS检测结合临床表现而最终确诊。

ADV是一种双链DNA病毒，可通过飞沫、粪-口以及血液传播，多致儿童急性呼吸道感染。但近年发现allo-HSCT等免疫低下人群可发生ADV感染。2019年Takamastsu等[3]个案报道1例脐血移植后患者发生ADV播散性感染（血液、颅内），最终死于多器官衰竭；该作者荟萃分析了26篇报道，共130例ADV感染病例，显示ADV可导致呼吸道及肺部感染、消化道感染/出血性肠炎、肝炎、膀胱炎、视网膜炎、脑膜炎等，病死率可达72％。国内孙于谦等[2]报道3例allo-HSCT后ADV感染，表现为ADV血症（PCR检测），其中1例合并ADV肠炎，3例均为Haplo-HSCT且合并GVHD。本研究中ADV的感染表现为：ADV血症、肝炎、脑炎、膀胱炎、肺炎。ADV可分为A～G共7型，其中ADV肺炎主要与ADV-B、C、E型感染有关，肝炎主要与C型有关，出血性膀胱炎与B型有关，肠炎与A、F型有关，结膜炎与B、D型有关等[12]。本研究中4例ADV-C型感染，ADV-B及D型各1例，其中以C型发生系统性感染多见，预后更差，这与文献[13]报道一致。

已报道的allo-HSCT后感染ADV危险因素有：无关供者移植或脐血移植、移植物去除T细胞、Ⅲ～Ⅳ度GVHD、外周血低淋巴细胞数（CD3^+^ T细胞<300个/ul）、应用ATG或抗CD25单抗等[14]。本研究中病例均具备至少2项上述危险因素。Sánchez-Céspedes等[15]曾报道61.1％（58/95）的allo-HSCT患者出现过短暂或持续的ADV血症，虽然部分患者未经干预，自行清除病毒，但结合国内现状，我们建议Haplo-HSCT患者作为ADV感染高危人群，有条件的单位可每周1次进行外周血ADV qPCR检测，评估抢先治疗时机，这也与欧洲白血病感染会议（ECIL）指南[7]较一致。此外儿童HSCT后ADV感染，病毒多经肠道活化，因此也有推荐基于粪便ADV的qPCR定期筛查结果进行抢先治疗[16]。有文献显示患者粪便ADV DNA高于10^6^拷贝数/g至出现ADV血症的中位时间为11 d[17]。

目前ADV感染的治疗包括尽快减停免疫抑制剂、抗病毒药物、免疫治疗、支持治疗等。CDV多被视为一线抗病毒药物，对各型ADV均有效。推荐剂量5 mg/kg，每周1次，连续2周ADV载量检测均阴性，可停用。Brincidofovir为CDV口服脂质体，有较高的抗病毒活性和较低肾毒性，可用于肾功异常或CDV疗效不佳患者。利巴韦林对ADV-C型部分有效，可与CDV联用。免疫治疗主要为DLI（多为低剂量）、ADV特异性细胞毒性T细胞输注等，用于药物疗效不佳者。此外，胸腺肽、IVIg、IL-7等药物也可应用。本研究中有5例接受CDV治疗，1例ADV载量转阴，临床好转，但除此例原发病复发死亡外，其余5例均对治疗无效，包括联合IVIg、胸腺肽、DLI等，均死于ADV感染，这也与文献显示的ADV感染高病死率一致[18]。
